# Peptide-Based Targeting of the L-Type Calcium Channel Corrects the Loss-of-Function Phenotype of Two Novel Mutations of the *CACNA1* Gene Associated With Brugada Syndrome

**DOI:** 10.3389/fphys.2020.616819

**Published:** 2021-01-08

**Authors:** Vittoria Di Mauro, Paola Ceriotti, Francesco Lodola, Nicolò Salvarani, Jessica Modica, Marie-Louise Bang, Andrea Mazzanti, Carlo Napolitano, Silvia G. Priori, Daniele Catalucci

**Affiliations:** ^1^ Institute of Genetic and Biomedical Research (IRGB), Milan Unit, National Research Council, Milan, Italy; ^2^ Humanitas Clinical and Research Center – IRCCS, Milan, Italy; ^3^ ICS Maugeri, IRCCS, Pavia, Italy; ^4^ Department of Biotechnology and Biosciences, University of Milano-Bicocca, Milan, Italy; ^5^ Department of Molecular Medicine, University of Pavia, Pavia, Italy

**Keywords:** brugada syndrome, arrhythmia, L-type calcium channel, mimetic peptide, channel trafficking, corrective therapy, cardiac disease

## Abstract

Brugada syndrome (BrS) is an inherited arrhythmogenic disease that may lead to sudden cardiac death in young adults with structurally normal hearts. No pharmacological therapy is available for BrS patients. This situation highlights the urgent need to overcome current difficulties by developing novel groundbreaking curative strategies. BrS has been associated with mutations in 18 different genes of which loss-of-function (LoF) *CACNA1C* mutations constitute the second most common cause. Here we tested the hypothesis that BrS associated with mutations in the *CACNA1C* gene encoding the L-type calcium channel (LTCC) pore-forming unit (Ca_v_α1.2) is functionally reverted by administration of a mimetic peptide (MP), which through binding to the LTCC chaperone beta subunit (Ca_v_β2) restores the physiological life cycle of aberrant LTCCs. Two novel Ca_v_α1.2 mutations associated with BrS were identified in young individuals. Transient transfection in heterologous and cardiac cells showed LoF phenotypes with reduced Ca^2+^ current (I_Ca_). In HEK293 cells overexpressing the two novel Ca_v_α1.2 mutations, Western blot analysis and cell surface biotinylation assays revealed reduced Ca_v_α1.2 protein levels at the plasma membrane for both mutants. Nano-BRET, Nano-Luciferase assays, and confocal microscopy analyses showed (i) reduced affinity of Ca_v_α1.2 for its Ca_v_β2 chaperone, (ii) shortened Ca_v_α1.2 half-life in the membrane, and (iii) impaired subcellular localization. Treatment of Ca_v_α1.2 mutant-transfected cells with a cell permeant MP restored channel trafficking and physiologic channel half-life, thereby resulting in I_Ca_ similar to wild type. These results represent the first step towards the development of a gene-specific treatment for BrS due to defective trafficking of mutant LTCC.

## Introduction

Brugada syndrome (BrS) is an inherited arrhythmogenic disorder causing sudden death in young individuals (Napolitano et al., 2012) and has been associated with mutations in 18 different genes. Loss-of-function (LoF) mutations in the *CACNA1C* and *CACNB2* genes encoding the alpha- (Ca_v_α1.2) and beta2- (Ca_v_β2) subunits of the L-type calcium channel (LTCC), respectively, account for up to 12% of genotyped BrS cases (Antzelevitch et al., 2007; Burashnikov et al., 2010; Napolitano and Antzelevitch, 2011).

The phenotypic consequences of mutations that are incompletely known and only in part experimentally reproduced in pharmacological models of LTCC LoF (Fish and Antzelevitch, 2004) are: (1) ST segment elevation due to a transmural voltage gradient, which is particularly accentuated in the right ventricular outflow tract epicardium that shows a prominent transient outward potassium current. In the precordial unipolar electrocardiographic leads V1 and V2 covering this region the typical Type I BrS pattern is detected; (2) QT shortening due to the reduction of the inward Ca^2+^ current (I_Ca_) in the ventricular myocardium (Burashnikov et al., 2010; Napolitano and Antzelevitch, 2011); (3) ST elevation in inferior or lateral leads, also called early repolarization (ERP; Burashnikov et al., 2010). All these conditions predispose to an increased risk of sudden death. The coexistence of ST segment elevation and abbreviated repolarization (short QT interval) often coexist in association with *CACNA1C* mutations and lead to an overlapping syndrome combining phenotypes of BrS and Short QT syndromes.

However, direct experimental assessments of the cellular consequences of LTCC-BrS mutants have been scanty and incomplete. In fact, the few Ca_v_α1.2 and Ca_v_β2 mutants that have been expressed in heterologous cellular systems mainly show a reduction in current density with absent or minor kinetic abnormalities (Antzelevitch et al., 2007; Bourdin et al., 2015). This effect is likely to be due to a reduced number of channels in the membrane that derives from either reduced forward trafficking or increased reverse trafficking/channel degradation, or both. However, detailed LTCC-mutant studies aimed at addressing the efficiency of both forward and reverse trafficking as well as their correction *via* novel molecular approaches have not yet been carried out.

In the clinical setting, risk stratification of BrS is relatively well defined (Priori et al., 2015), while the therapeutic options are limited to the use of an implantable cardioverter defibrillator (ICD), which may hamper the quality of life of young patients due to inappropriate shocks or lead-related complications that occur in up to 30% of patients (Napolitano et al., 2012). No pharmacological therapy has been demonstrated to be able to reduce life threatening arrhythmic events in controlled clinical trials. Thus, in this context, the identification of an innovative antiarrhythmic therapy would represent a major step forward with remarkable clinical impact.

Our group has developed an LTCC-specific peptide (MP), which can restore physiological LTCC levels when the channel density at the plasma membrane is reduced (Rusconi et al., 2016; Miragoli et al., 2018; Romanelli et al., 2018). The MP, designed from the C-terminal tail of the otherwise globular Ca_v_β2, binds to a region (Tail Interacting Domain, TID; Rusconi et al., 2016) within the globular Ca_v_β2 chaperone, facilitating the restoration of Ca_v_α1.2 density at the plasma membrane. Therefore, the MP directly acts the regulation of LTCC intracellular protein trafficking, i.e., reducing Ca_v_α1.2 reverse trafficking and protein degradation by preventing LTCC endocytosis as well as promoting Ca_v_α1.2 forward trafficking by facilitating efficient Ca_v_β2-mediated chaperoning of Ca_v_α1.2 to the plasma membrane. Importantly, this effect does not come at the cost of altered contractility or pro-arrhythmic effects as the gating kinetics of the LTCC remain unaltered following MP administration both *in vitro* and *in vivo* (Rusconi et al., 2016; Miragoli et al., 2018). The MP, designed with an arginine-rich cationic cell-penetrating peptide (R7W-MP; Rusconi et al., 2016) or loaded in inhalable nanoparticles (Miragoli et al., 2018), was shown by our group to be effective in an *in vivo* model of diabetic cardiomyopathy (Rusconi et al., 2016; Miragoli et al., 2018).

Based on these previous results, we hypothesized that the MP could correct electrophysiological and molecular defects associated with LoF LTCC mutations linked to BrS. Two novel Ca_v_α1.2 mutations (T320M and Q428E) were identified in two young individuals with a diagnosis of BrS. Electrophysiological and molecular characterization showed a LoF I_Ca_ phenotype associated with reduced LTCC membrane localization. In this setting, we evaluated the pharmacological application of R7W-MP and demonstrated that treatment of heterologous and cardiac cells carrying the two mutations is sufficient to correct the LTTC channel density at the plasma membrane and restore physiological I_Ca_.

## Materials and Methods

### Genetic Analysis

Gene mutations were identified in subjects with BrS enrolled in the TRIAD registry of inherited arrhythmias at the ICS Maugeri Institute.^1^ Genetic analysis was performed by conventional Sanger sequencing and included the entire open reading frame of *CACNA1C* (NM_000719), including 8 alternative exons (LRG_334). The coding region of *SCN5A*, the most common gene associated with BrS (Crotti et al., 2012) was also screened (NM_198056.29). The diagnosis of BrS was established in agreement with the current accepted criteria (Priori et al., 2015) after excluding the presence of structural cardiomyopathies. The TRIAD registry (registration number 911CEC, 22nd May 2013) is approved by the Ethical Committee of the ICS Maugeri, Pavia, Italy, and the study conforms to the principles outlined in the Declaration of Helsinki. All patients gave written informed consent agreeing to the use of their clinical and genetic data for research purposes.

### DNA Constructs and Peptides

Site-directed mutagenesis was performed on full-length human alternative transcript variant 17 *CACNA1C* (NM_001129843) cloned into the pCMV6-XL4 vector (Origene Technologies) using the QuickChange II XL kit (Agilent Technologies). Human *CACN2B* (NM_201572) was cloned into the pCMV6-XL5 vector (Origene Technologies). For the Nano-Luciferase assay, WT and mutant Ca_v_α1.2 cDNAs were cloned into the pNLF1-N vector (Promega), whereas for the BRET assay, Ca_v_α1.2 and Ca_v_β2 cDNAs were cloned into the pNLF1-N and HaloTag-pFN21A vectors, respectively. All clonings were performed using the In-fusion HD Cloning Plus kit (Clontech). All constructs were verified by sequencing of the complete insert. Peptides were synthetized by GenScript (USA). R7W-MP: RRRRRRRW-DQRPDREAPRS; R7W-Scr: RRRRRRRW-DQPPSRRDERA.

### Cell Culture Conditions and Transfection

Human embryonic kidney cells (HEK293) were cultured in DMEM (Sigma) supplemented with 10% HI-FBS (Life Technologies), 100 U/ml penicillin, and 0.1 mg/ml streptomycin (Euroclone). HL-1 cardiac cells were maintained in a special culture medium according to an optimized protocol provided by Dr. Claycomb’s laboratory. HEK293 and HL-1 cells were transiently transfected with Ca_v_β2 and Ca_v_α1.2 (WT or mutant). Viafect (Promega) and Lipofectamine (ThermoFisher) transfection reagent were used for HL-1 and HEK293 cells, respectively.

### Electrophysiology

L-type Ca^2+^ (I_Ca,L_) currents were measured using the Patch-Clamp technique in the whole cell configuration and voltage-clamp mode. For the experiments performed in HEK293 cells the extracellular solution had the following composition (in mM): 140 NaCl, 2 CaCl_2_, 1 MgCl_2_, 10 HEPES, and 5 Glucose, pH 7.4 adjusted with NaOH. Patch pipettes were pulled from borosilicate glass (WPI, Inc) on a P-97 horizontal puller (Sutter Instruments) and had a resistance of 2 to 3 MΩ when filled with a solution containing (in mmol/L): 120 CsCl, 2 MgCl_2_, 10 HEPES, 5 CaCl_2_, 2 MgATP, and 10 EGTA, pH 7.2 adjusted with CsOH. Recording protocols were applied by 300 ms depolarizing pulses from a holding potential of −80 mV with 10 mV steps from −60 mV to +50 mV. Membrane currents were analyzed with pCLAMP 9.2 (Axon Instruments) and Origin 6.0 (Northampton, USA) software. All the experiments were performed at room temperature (24–26°C). For HL-1 cells all measurements were carried out at 36 ± 0.5°C and I_Ca,L_ was recorded in extracellular and patch pipette solutions where potassium currents were suppressed by substituting K^+^ with Cs^+^. The composition of the extracellular solution was (mmol/l): NaCl, 140; CsCl, 5.4; MgCl_2_, 1.25; CaCl_2_, 2; HEPES, 10; D-glucose, 10; adjusted to pH 7.4 with NaOH; while the patch pipette solution contained (mmol/l): CsCl, 120; EGTA, 10; MgCl_2_, 4; HEPES, 10; 4 Na_2_ATP, 4; adjusted to pH 7.4 with CsOH. Experimental protocols were controlled using Clampex software (version 10.3 of pClamp, Axon Instruments). Pipette resistances ranged from 2 to 3 MOhm and pipette potentials were zeroed before cell contact. Peak I_Ca,L_ was measured in response to 400 ms depolarization steps ranging from −50 to 70 mV at a rate of 0.33 Hz (holding potential of −90 mV). A 50-ms prepulse to −40 mV was used to inactivate sodium channels and T-type I_Ca_ (if present). In both cells models, steady state activation curves were derived from each I-V relation and described by fitting experimental points with the Boltzmann equation. For all the experimental settings, 1.3 μM R7W-MP or R7W-Scr was added to the medium 24 h before the analysis.

### Western Blot Analyses

Protein expression was evaluated by Western blot analyses according to standard procedures and as previously described (Rusconi et al., 2016). 3–8% NuPAGE Tris-Acetate gels (Life Technologies) were used for electrophoresis followed by blotting to a PVDF membrane (Millipore). The following antibodies were used: Ca_v_α1.2 and Transferrin Receptor (Abcam); GAPDH (14C10) (Cell Signaling Technology); Goat anti-mouse-HRP and Goat anti-rabbit-HRP (Thermo Fisher Scientific). ECL was used for protein detection using a Chemidoc MP Imaging System (Biorad).

### Cell Surface Biotinylation

The assay was performed as previously described (Rusconi et al., 2016). Briefly, transfected HEK293 cells were washed in ice cold PBS, biotinylated for 20 min with 0.5 mg/ml EZ Link Sulfo-NHS-LC-Biotin (Thermo Scientific), and subsequently lysed in RIPA buffer. After incubation with 50 μl High Capacity NeutrAvidin agarose beads (Thermo Scientific), biotinylated membrane proteins trapped to NeutrAvidin beads were washed and assayed by Western blot analysis for Ca_v_α1.2 (Abcam).

Protein level detection for Transferrin Receptor (plasma membrane fraction) and GAPDH (cytoplasm fraction) were used as positive and negative controls, respectively, for quality assessment of the assay.

### Nano Luciferase Assay

The NanoLuc Luciferase assay was performed as previously described (Rusconi et al., 2016). Briefly, HEK293 cells pretreated for 30 min with 20 μM cycloheximide were transfected and time-course analysis (NanoLuc Luciferase activity from 0.5 to 8 h post treatment) was performed using a Synergy 4 instrument (BioTek) as described by the manufacturer (Promega).

### Nano-BRET Assay

The NanoBRET Assay was performed as previously described (Rusconi et al., 2016). Briefly, transfected HEK293 cells were treated with 100 nM NanoBRET 618 Ligand (Promega), and signals were detected 6 h after treatment. Signals were detected using a Synergy 4 instrument (BioTek).

### Confocal Microscopy

Transfected HEK293 cells were blocked and permeabilized with 3% normal goat serum, 0.1% Triton X-100, and 50 mM glycine in 1 × PBS for 1 h. After incubation with Ca_v_α1.2 primary antibody and secondary antibodies, cells were scanned with an Olympus FluoView FV1000 confocal laser-scanning microscope. Images were analyzed using Fiji Image J software (National Institutes of Health). Measurements of the relative increase in plasma membrane localization were performed as described elsewhere (Viard et al., 2004). Internalization was detected by calculation of cytoplasm fluorescence (Fc)/membrane fluorescence (Fm).

### Statistical Analysis

Data are presented as mean ± standard error of the mean (SEM) or as individual values. Assessment of the normality of the data was calculated using the Kolmogorov-Smirnov (K-S) test. Statistical comparison between two samples was performed in at least three independent experiments with the Mann-Whitney *T* test. For multiple confrontations, one-way ANOVA followed by Tukey’s *post hoc* test or the Kruskal-Wallis test in combination with Dunn’s test were applied.

Prism 6.0 software (GraphPad Software, CA) was used for result analyses and statistical calculation. *p* < 0.05 was considered statistically significant.

## Results

### Brugada Patients With Novel Ca_v_α1.2 Mutations

**Figure 1:**
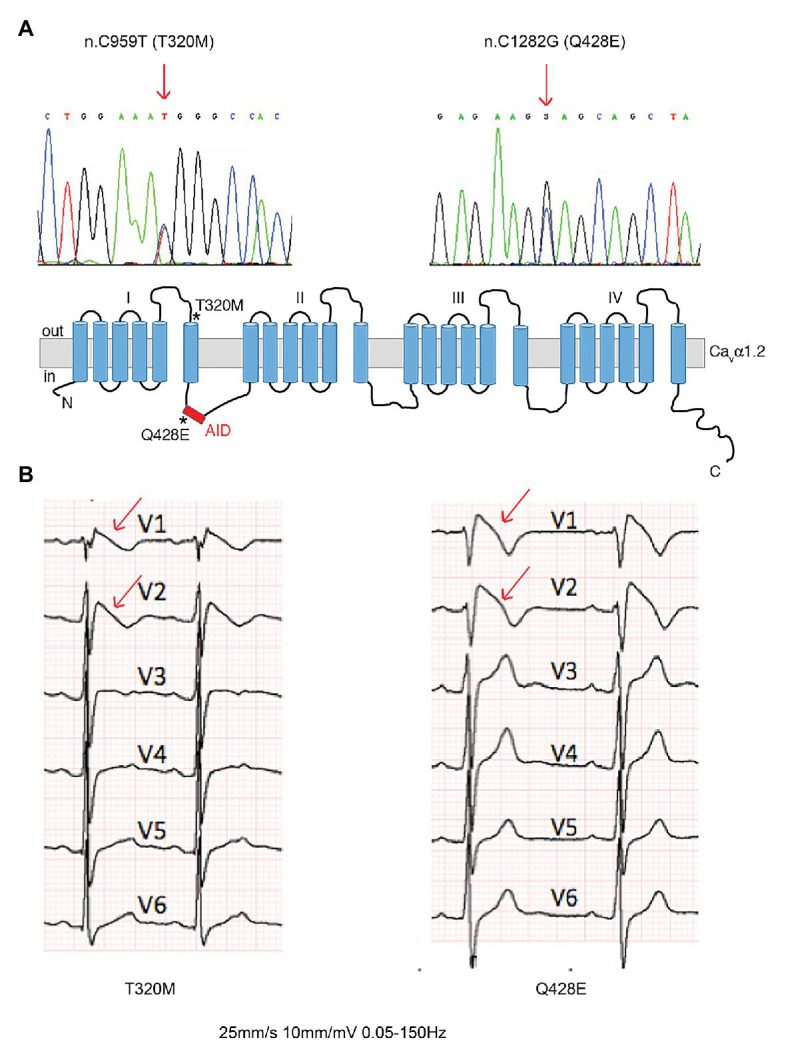
Brugada patients with novel Ca_v_α1.2 loss-of-function (LoF) mutations. **(A)** DNA sequence analysis showing the two heterozygous single nucleotide substitutions (arrows) in Ca_v_α1.2 responsible for the T320M and Q428E missense mutations, and a schematic representation of Ca_v_α1.2, indicating the location (stars) of mutants. **(B)** Baseline ECGs showing type 1 coved ST segment elevation pattern (arrows) in the two patients. AID, alpha-interaction domain.

Two Ca_v_α1.2 mutations (n.C959T/p.T320M and n.C1282G/p.Q428E) were identified by genetic screening of subjects with clinical diagnosis of BrS and referred to our Center for genetic testing. Both mutants mapped in highly conserved regions between domains I and II of Ca_v_α1.2 (Figure 1A).

The Ca_v_α1.2 T320M mutation was identified in a 33-year-old asymptomatic male patient, who was admitted at the emergency room for abdominal pain. The electrocardiogram (ECG) showed diagnostic BrS features (Figure 1B, left panel). The patient has remained asymptomatic for the following 8 years of follow-up, showing spontaneous manifestation of a type 1 pattern during 24-h Holter recordings.

The Ca_v_α1.2 Q428E mutation was identified in a 32-year-old male diagnosed after a cardiac arrest occurring at rest, showing a spontaneous type 1 BrS pattern at baseline ECG (Figure 1B, right panel). The patient received an ICD. After 13 years of follow-up, ICD memory interrogation revealed two episodes of self-terminating polymorphic ventricular tachycardia and runs of atrial fibrillation.

### A Loss-of-Function Phenotype Is Associated With the T320M and Q428E Ca_v_α1.2 Mutations

**Figure 2:**
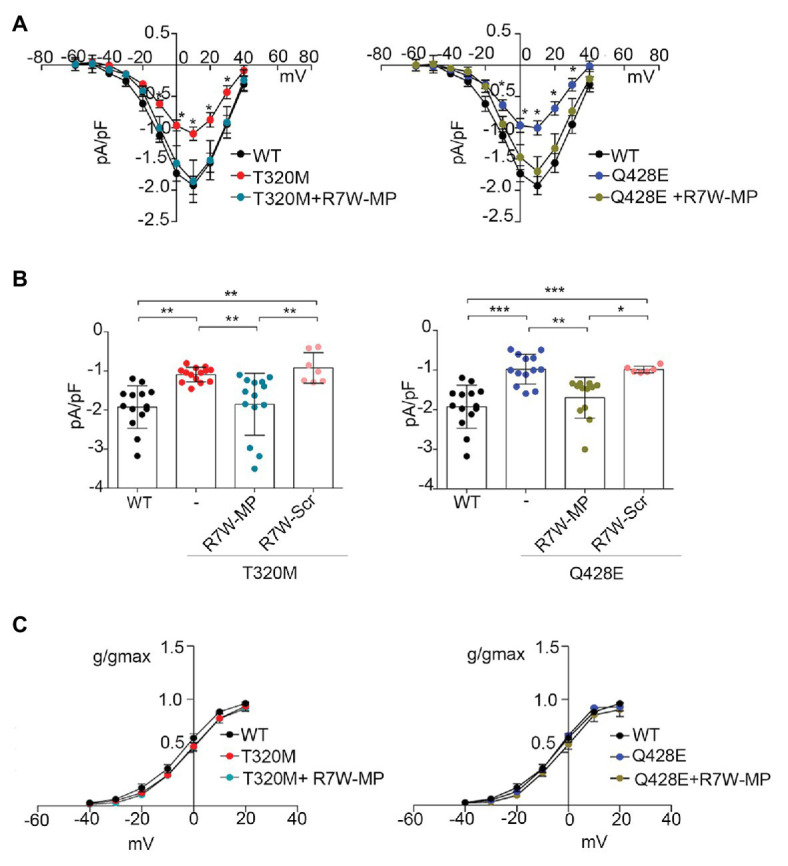
Treatment with R7W-MP corrects Ca^2+^ alterations due to Ca_v_α1.2 mutations in a heterologous cell model. **(A)** Current-voltage (I-V) relationships recorded in HEK293 cells transiently co-transfected with Ca_v_α1.2 WT (WT, *n* = 14), Ca_v_α1.2 T320M (T320M, *n* = 14), or Ca_v_α1.2 Q428E (Q428E, *n* = 13) and Ca_v_β2 at baseline and 24 h after treatment with 1.3 μM R7W-MP (T320M + R7W-MP, *n* = 14; Q428E + R7W-MP, *n* = 12). **(B)** Single dots and histograms (average) showing I_Ca_ measured at a test pulse of +10 mV for all the above-mentioned experimental conditions. ^*^ *p* < 0.05, ^**^ *p* < 0.01, ^***^ *p* < 0.001 (Kruskal-Wallis test). **(C)** Graphical representation of I_Ca_ activation-curves (g/gmax) for WT (V_1/2_ = −4.09 ± 1.81, slope = 8.29 ± 0.37), T320M (V_1/2_ = −1.90 ± 0.93, slope = 8.79 ± 0.60), and Q428E (V_1/2_ = −5.10 ± 0.92, slope = 7.64 ± 0.95). Kinetic curves did not change after R7W-MP treatment (V_1/2_ = −2.19 ± 1.18, slope = 8.21 ± 0.37; V_1/2_ = −3.69 ± 0.64, slope = 7.71 ± 0.31 for T320M + R7W-MP and Q428E + R7W-MP, respectively).

As a first step, cDNAs of WT, T320M, and Q428E Ca_v_α1.2 as well as of the accessory Ca_v_ß2 subunit were cloned in expression vectors, which were cotransfected in heterologous HEK293 and cardiac HL-1 cells for assessment of the electrophysiological phenotype. In HEK293 cells, patch clamp measurements at test pulse +10 mV showed an about 50% reduction in I_Ca_ currents in cells transfected with Ca_v_α1.2 T320M (−1.09 ± 0.05 pA/pF; *n* = 14; *p* < 0.01) and Q428E (−0.98 ± 0.10 pA/pF; *n* = 13; *p* < 0.001) compared to WT transfected cells (−1.92 ± 0.15 pA/pF; *n* = 14; Figures 2A,B).

Experiments were also performed in HL-1 cells where, due to the presence of an endogenous WT Ca_v_α1.2 subunit, we were able to evaluate the effect of the mutant channel in conditions of heterozygosity similar to those present in BrS patients. Data showed a reduction of I_Ca_ in both Ca_v_α1.2 T320M- (−6.73 ± 0.76 pA/pF; *n* = 7; *p* < 0.05) and Q428E- (−6.11 ± 0.73 pA/pF; *n* = 6; *p* < 0.01) transfected cells compared to Ca_v_α1.2 WT-transfected cells (−9.12 ± 0.60 pA/pF; *n* = 11; Supplementary Figures 1A,B). Altogether, these data provide evidence of a LoF phenotype for both T320M and Q428E Ca_v_α1.2 mutations.

### R7W-MP Restores Ca^2+^ Current Reduction Due to Ca_v_α1.2 Loss-of-Function Mutations

Based on our recent results showing the ability of nanoparticle-loaded MP (Miragoli et al., 2018) or cell permeant R7W-MP (Rusconi et al., 2016) to recover LTCC dysregulation in diabetic cardiomyopathy, we hypothesized that also in the case of T320M and Q428E LoF BrS, the MP therapeutic peptide can correct the LTCC abnormalities. To test this, a similar experiment was performed as above, and HEK293-transfected cells were evaluated for I_Ca_ 24 h after R7W-MP-treatment. As shown in Figures 2A,B, I_Ca_ currents in R7W-MP-treated HEK293 cells cotransfected with Ca_v_α1.2 T320M (−1.85 ± 0.21 pA/pF; *n* = 14) or Q428E (−1.70 ± 0.15 pA/pF; *n* = 12) and the Ca_v_ß2 subunit were restored to levels similar to those of Ca_v_α1.2 WT-transfected cells. Notably, no kinetic abnormalities were observed either before or after R7W-MP-treatment (Figure 2C), consistent with our previous study (Rusconi et al., 2016). Furthermore, no effects were obtained following treatment with R7W-scramble peptide (R7W-Scr) either in Ca_v_α1.2 T320M- (−0.92 ± 0.15 pA/pF; *n* = 7) or Q428E- (−0.98 ± 0.03 pA/pF; *n* = 7) transfected cells (Figure 2B).

Notably, a similar R7W-MP-dependent corrective effect was also observed in HL-1 cardiac cells, where I_Ca_ currents in both Ca_v_α1.2 T320M- (−9.12 ± 0.60 pA/pF; *n* = 12) and Q428E- (−9.31 ± 0.65 pA/pF; *n* = 8) transfected cells were restored to levels comparable to non-transfected cells without affecting channel kinetics (Supplementary Figure 1). On the other hand, no positive modulation of I_Ca_ currents was observed in HL-1 cells expressing Ca_v_α1.2 T320M (−6.34 ± 0.80 pA/pF, *n* = 9) or Q428E (−5.97 ± 1.06 pA/pF, *n* = 6) when the R7W-Scr was used (Supplementary Figure 1B).

Altogether these results showed that, without affecting channel gating properties, R7W-MP can correct the T320M and Q428E LoF phenotype in both cardiac and heterologous cells. Based on this evidence and with the attempt to avoid any experimental bias due to the endogenous Ca_v_α1.2, all further analyses dissecting the molecular mechanisms underlying the LoF mutant-dependent deregulation of LTCCs and the corrective effect of R7W-MP were subsequently performed only within the heterologous HEK293 cells.

### R7W-MP Corrects the Reduced Interaction Between Mutant Ca_v_α1.2 and Ca_v_β2

**Figure 3:**
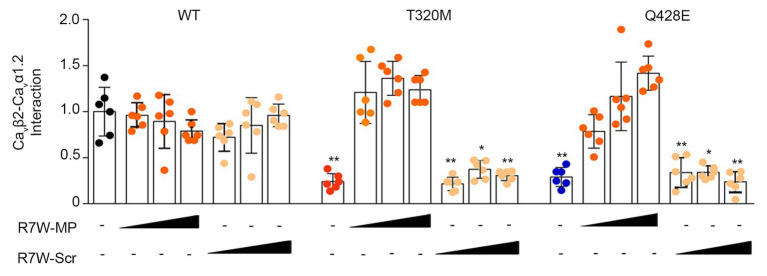
R7W-MP corrects the reduced interaction between mutant Ca_v_α1.2 and Ca_v_β2. Ca_v_β2-Ca_v_α1.2 protein affinity as measured in a BRET assay in HEK293 cells transfected with Ca_v_β2-NanoLuc and Ca_v_α1.2-Halo (WT, T320M, and Q428E mutant) and treated with increasing doses (0.12, 1.3, and 10.2 μM) of R7W-MP and R7W-Scr as indicated (*n* = 6). ^*^ *p* < 0.05; ^**^ *p* < 0.01 (Mann-Whitney test). Black, red, and blue indicate untreated conditions.

Point mutations in the I-II loop of the Ca_v_α1.2 pore unit have previously been shown to disrupt its interaction with Ca_v_β2 and prevent its targeting to the plasma membrane (Simms and Zamponi, 2012). Based on the localization of the Ca_v_α1.2 T320M and Q428E variants in proximity to and inside the α-interacting domain (AID), respectively (Figure 1A), we hypothesized that these mutations affect the Ca_v_β2 - Ca_v_α1.2 interaction and possibly related intracellular trafficking features. To assess this hypothesis, we performed a bioluminescence resonance energy transfer (BRET) assay and found that both mutations strongly reduce the Ca_v_β2-Ca_v_α1.2 interaction (Ca_v_α1.2 T320M by 80% and Ca_v_α1.2 Q428E by 70%) (Figure 3). Importantly, R7W-MP-administration corrected the defective interaction resulting in full recovery, while R7W-Scr-administration had no effect (Figure 3).

This result supports the hypothesis that R7W-MP-treatment facilitates I_Ca_ recovery *via* restoration of Ca_v_α1.2 trafficking towards the plasma membrane.

### R7W-MP Restores LTCC Protein Density at the Plasma Membrane in Ca_v_α1.2 Mutant-Transfected Cells

**Figure 4:**
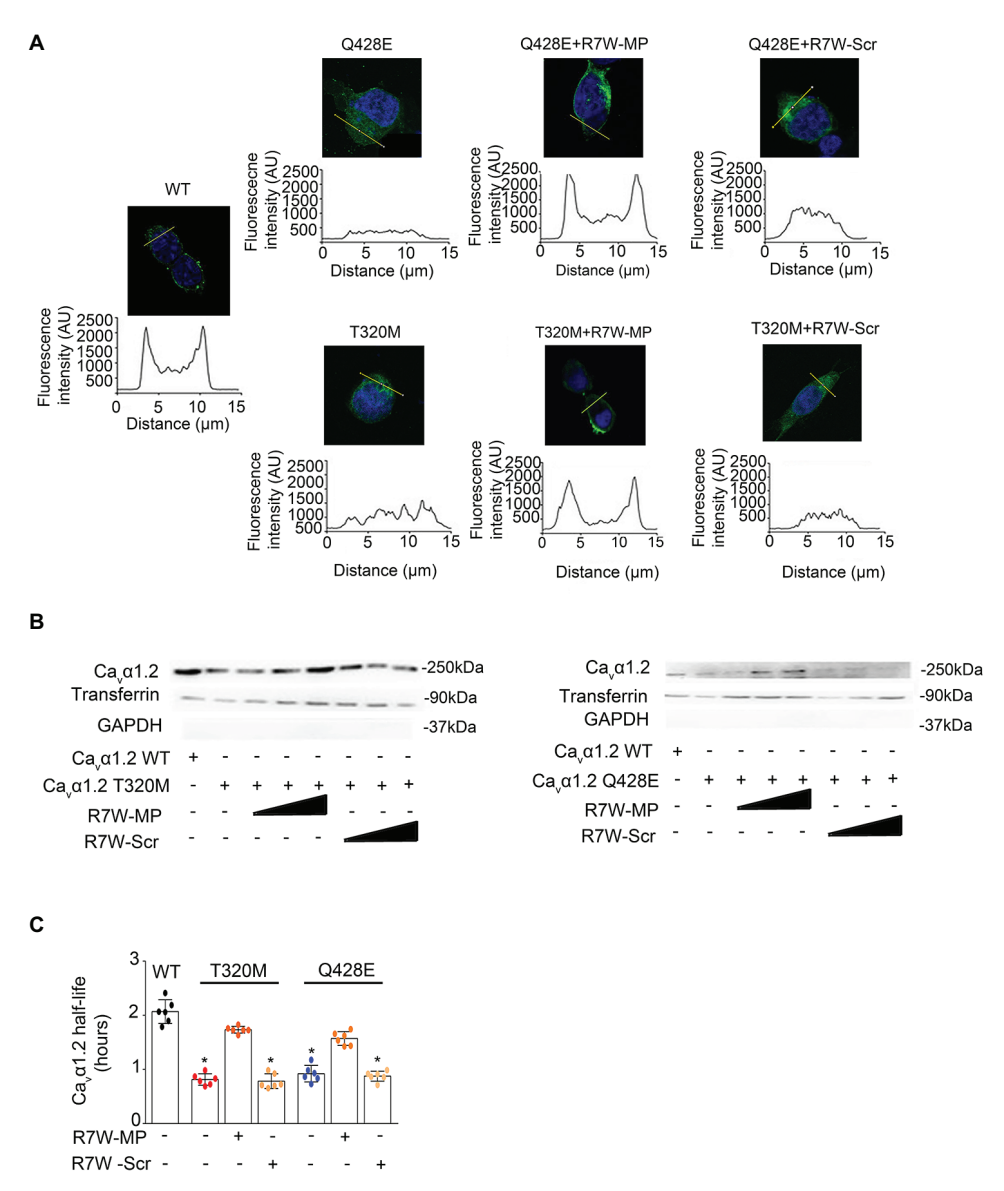
R7W-MP corrects LTCC density at the plasma membrane in Ca_v_α1.2 mutant-transfected cells. **(A)** Subcellular localization of Ca_v_α1.2 (top) and line scan analyses (bottom) for each condition as indicated. AU, arbitrary units. Scale bars (in white), 10 μm (*n* = 50). Representative experiments are shown. Cells were treated with increasing doses (0.12, 1.3, and 10.2 μM) of R7W-MP or R7W-Scr. **(B)** Cell surface biotinylation assay followed by followed by Western blot analysis (a representative image is shown) on transfected cells treated as indicated (*n* = 3). **(C)** Ca_v_α1.2 half-life as measured in a NanoLuc luciferase assay. HEK293 cells were transfected with Ca_v_α1.2-NanoLuc (WT, T320M, and Q428E mutant) and treated with 1.3 μM R7W-MP or R7W-Scr as indicated (*n* = 6). ^*^ *p* < 0.05 (Mann-Whitney test).

Alterations in the interaction between Ca_v_β2 and Ca_v_α1.2 have previously been shown to affect the relative distribution of Ca_v_α1.2 between the plasma membrane and the cytosol (Viard et al., 2004). Thus, to determine the effect of the Ca_v_α1.2 mutations on Ca_v_β2 trafficking, we measured fluorescence intensity profiles in Ca_v_α1.2 WT- and mutant-transfected cells immunostained for Ca_v_α1.2. Whereas intensity profiles with predominant fluorescence at the plasma membrane were observed in Ca_v_α1.2 WT-transfected cells, as expected (Viard et al., 2004), cells transfected with the two Ca_v_α1.2 mutants showed homogeneous staining throughout the cell, demonstrating a clear trafficking defect (Figure 4A and Supplementary Figure 2). This was further supported by a surface protein biotinylation assay, which showed a reduction in Ca_v_α1.2 plasma membrane levels in cells transfected with Ca_v_α1.2 T320M (66%) and Q428E (62%) compared to WT (Figure 4B). These results are consistent with the patch-clamp data, showing reduced I_Ca_ in Ca_v_α1.2 mutant-transfected cells (Figure 2 and Supplementary Figure 1).

Notably, treatment with R7W-MP fully restored the proper plasma membrane localization of both Ca_v_α1.2 T320M and Q428E mutants, resulting in fluorescence intensity profiles, comparable to those of Ca_v_α1.2 WT-transfected cells (Figure 4A and Supplementary Figure 2A). Consistently, a trend of recovery of Ca_v_α1.2 T320M (81%) and Q428E (84%) membrane densities was obtained in R7W-MP-treated cells compared to R7W-Scr-treated cells (Figure 4B and Supplementary Figure 2B). Taken together, these data demonstrate that the R7W-MP can efficiently correct the trafficking defect of Ca_v_α1.2 mutants.

Finally, due to the overall reduction in LTCC density, a decreased half-life of the channel might be expected. To determine this, we performed a nano-luciferase protein stability assay, which consistent with our hypothesis, revealed a 61 and 55% reduction in Ca_v_α1.2 half-life for the T320M and Q428E mutants compared to WT, respectively (Figure 4C). R7W-MP-treatment largely restored the reduced Ca_v_α1.2 half-life, demonstrating that the R7W-MP also has a corrective effect on Ca_v_α1.2 half-life.

## Discussion

The prevalence of LoF *CACNA1C* mutations that cause ECG abnormalities and risk of sudden death is not yet fully established and its definition is hampered by the complexity of the associated clinical phenotypes. Indeed, they have not only been associated with ST elevation in the right precordial leads (i.e., Brugada ECG pattern) but also with ERP and unexplained sudden death in the normal heart (also called “idiopathic ventricular fibrillation”; Priori and Napolitano, 2018). In this context, the Ca_v_α1.2 T320M mutation reported in this paper has a very low allelic frequency in the population (0.0007% GnomAD database) and it has been observed in one subject from a cohort of 174 cases with unexplained cardiac arrest from the CASPER registry (Mellor et al., 2017). The Ca_v_α1.2 Q428E mutation has never been observed, nor it is present in the population genomic databases. Both patients presented with typical Brugada type 1 ECG and mild intraventricular conduction delay. The Q428E carrier also presented J point elevation (ERP) in the inferior leads.

The percentage of *CACNA1C* mutations that cause a trafficking defect and that can be rescued is clinically relevant since other reported mutations display impaired trafficking (Antzelevitch et al., 2007). Furthermore the fact that many mutations characterized *in vitro* (Fish and Antzelevitch, 2004; Antzelevitch et al., 2007; Bourdin et al., 2015; Sutphin et al., 2016) cause a loss of current density in the absence of kinetic abnormalities strongly suggest the idea that a trafficking defect is often implicated.

This proof of concept study demonstrates for the first time that it is possible to design therapeutic molecules able to correct the deficient trafficking of mutant LoF Ca_v_α1.2, thereby restoring I_Ca_ to WT levels. Although only few LTCC mutations associated with BrS have been functionally characterized so far, it is well established that LoF pathogenic variants cause a reduction in current density (Antzelevitch et al., 2007; Burashnikov et al., 2010; Bourdin et al., 2015), often secondary to a trafficking defect (Antzelevitch et al., 2007; Burashnikov et al., 2010; Bourdin et al., 2015). This mechanism was also found to be responsible for the pathological effect of the two new mutations reported in this study, where impaired forward trafficking and increased protein turnover (reduced half-life) were observed. Notably, these molecular abnormalities were corrected by R7W-MP treatment.

The therapy of BrS is currently based mainly on the use of an ICD, which is associated with various complications, especially when used in young subjects (Miyazaki et al., 2013; Olde Nordkamp et al., 2016). Quinidine has been proposed for patients with contraindications to ICD or to treat electrical storms (Priori et al., 2015). However, its efficacy to prevent cardiac events has not been definitely established and the high incidence of side effects often leads to therapy withdrawal.

Therapeutic peptides are increasingly recognized for their high selectivity and efficiency for the treatment of various diseases (Fosgerau and Hoffmann, 2015). Several are currently under evaluation in clinical trials (~200 peptide drugs in clinical development) and >60 peptide medicines are already approved in the US market. Semaglutide, a peptide-based analog to the human glucagon-like peptide-1, is a recently approved drug for the treatment of type 2 diabetes (Bucheit et al., 2020). Here we tested the hypothesis that our Ca_v_β2-targeting R7W-MP, regulating the subcellular distribution and life cycle of Ca_v_α1.2 (Rusconi et al., 2016) can be used to selectively modulate cardiac electrophysiology with a demonstrable therapeutic effect. In particular, treatment of Ca_v_α1.2 mutant-transfected cells with R7W-MP restored the interaction between Ca_v_α1.2 and Ca_v_β2, correcting Ca_v_α1.2 targeting to the plasma membrane through normalization of deficient trafficking and increased Ca_v_α1.2 half-life. These molecular effects lead to a reversal of the electrophysiological abnormalities associated with the Ca_v_α1.2 LoF mutants in both heterologous and cardiac cells. It is particular encouraging that the R7W-MP was not found to alter channel gating properties in agreement with our previous studies, where we also showed that R7W-MP or nanoparticle-loaded MP do not affect cardiomyocyte contractility and Ca^2+^ handling, or have pro-arrhythmogenic effects in a structurally normal heart (Rusconi et al., 2016; Miragoli et al., 2018). In contrast, previously identified Ca^2+^ agonists (e.g., BAYK8644) have been associated with unwanted changes in LTCC gating kinetics and altered Ca^2+^ release from the sarcoplasmic reticulum (Katoh et al., 2000), causing deleterious effects. The future availability of *in vivo* models accurately replicating the typical transmural heterogeneity of action potential duration and conduction velocity of BrS (Zhang et al., 2015) will be critical for further exploration towards translation to the clinic.

Altogether, we here prove that R7W-MP-treatment is sufficient for correction of defective Ca_v_α1.2 trafficking associated with BrS type 3, which may open a new perspective for the treatment of at least some of the genetic defects causing BrS. Whether or not the same effect can be achieved in the treatment of other LTCC LoF mutants (such as those found in the C-terminal region of Ca_v_α1.2) should be the object of future studies.

In summary, our data provide the evidence that R7W-MP, by controlling the LTCC life cycle, can be used to treat the molecular dysfunctions and electrophysiological consequences of trafficking-defective *CACNA1C* mutations associated with BrS. To the best of our knowledge the MP mechanism of action represents the first example of an LTCC-targeting therapeutic molecule that can correct I_Ca_ defects through modulation of channel density at the plasma membrane. At a broader perspective, it is conceivable to think that MP in combination with safe and cardiac-specific carriers [e.g., inhalable nanoparticles (Di Mauro et al., 2016; Miragoli et al., 2018) or cell-specific targeting aptamers (Thiel et al., 2016; Romanelli et al., 2018)], could lead to the development of a new therapy for LTCC-related BrS, a severe arrhythmogenic disorder for which limited therapeutic options are currently available.

## Data Availability Statement

The raw data supporting the conclusions of this article will be made available by the authors, without undue reservation.

## Author Contributions

VDM, PC, FL, JM, NS, and AM performed the experiment and analyzed data. VDM, FL, NS, MLB, SP, and DC wrote the manuscript. DC and SP designed the study. DC and SP acquired the funding for the study. All authors contributed to the article and approved the submitted version.

### Conflict of Interest

The authors declare that the research was conducted in the absence of any commercial or financial relationships that could be construed as a potential conflict of interest.

## Footnotes

^1^http://triad.fsm.it/triadweb/

## Supplementary Material

SUPPLEMENTARY FIGURE 1 Treatment with R7W-MP corrects Ca^2+^ alterations due to Ca_v_α1.2 mutations in a cardiac cell model. **(A)** Current-voltage (I-V) relationships recorded in non-transfected HL-1 cells (WT, *n* = 11) or cells transiently transfected with Ca_v_α1.2 T320M (T320M, *n* = 7) or Ca_v_α1.2 Q428E (Q428E, *n* = 6) at baseline and 24 h after treatment with 1.3 μM R7W-MP (T320M + R7W-MP, *n* = 12; Q428E + R7W-MP, *n* = 8). **(B)** Single dots and histograms (average) are showing I_Ca_ measured at a test pulse of +10 mV for all the above-mentioned experimental conditions. ^*^*p* < 0.05, ^**^*p* < 0.01 (Kruskal-Wallis test). **(C)** Graphical representation of I_Ca_ activation-curves (g/gmax) for WT (V_1/2_ = −10.34 ± 1.28, slope = 7.08 ± 0.57), T320M (V_1/2_ = −7.08 ± 1.77, slope = 7.29 ± 0.46), and Q428E (V_1/2_ = −7.10 ± 1.64, slope = 6.59 ± 0.25). Kinetic curves did not change after R7W-MP treatment (V_1/2_ = −8.28 ± 1.32 and slope = 6.78 ± 0.28; V_1/2_ = −6.91 ± 0.91 and slope = 6.66 ± 0.37 for T320M + R7W-MP and Q428E + R7W-MP, respectively).

SUPPLEMENTARY FIGURE 2 R7W-MP corrects LTCC density at the plasma membrane in Ca_v_α1.2 mutant-transfected HEK293 cells. **(A)** Quantification of the fluorescence signal in the cytoplasm and the membrane of the cells (*n* = 15) in Figure 4A. Fc, cytoplasmic fluorescence; Fm, membrane fluorescence. ^***^ *p* < 0.001 (one-way ANOVA). **(B)** Graphical representation of densitometry of Western blot for Ca_v_α1.2 in the cell surface biotinylation assays in Figure 4B. (Kruskal-Wallis test).
