# Supplementary figures and images for: Peptide-Based Targeting of the L-Type Calcium Channel Corrects the Loss-of-Function Phenotype of Two Novel Mutations of the CACNA1 Gene Associated With Brugada Syndrome
Source: Front Physiol. 2021 Jan 8;11:616819. doi: 10.3389/fphys.2020.616819 (PMC7821386; doi:10.3389/fphys.2020.616819)

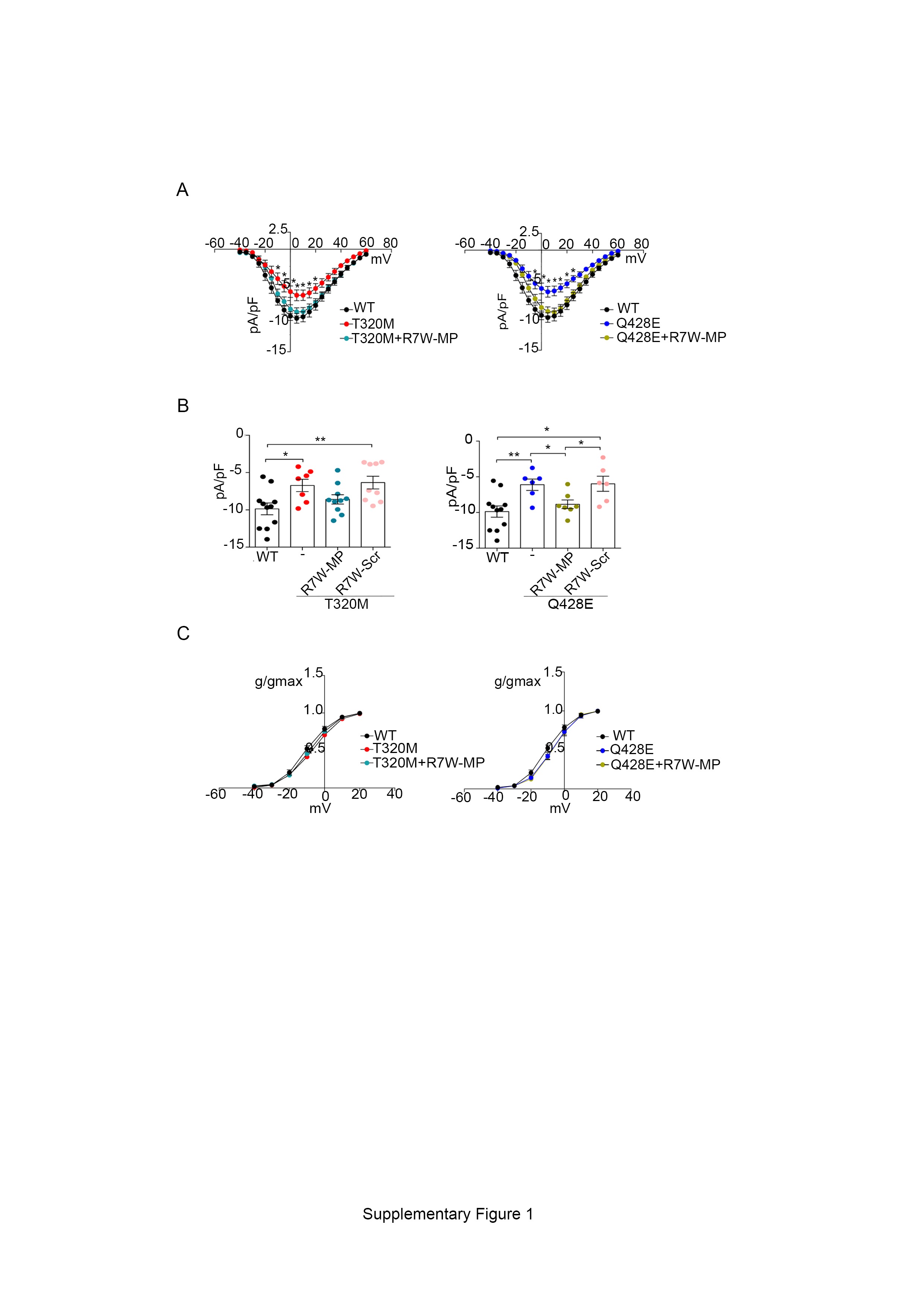

Supplement: SUPPLEMENTARY FIGURE 1 — Treatment with R7W-MP corrects Ca2+ alterations due to Cavα1.2 mutations in a cardiac cell model. (A) Current-voltage (I-V) relationships recorded in non-transfected HL-1 cells (WT, n = 11) or cells transiently transfected with Cavα1.2 T320M (T320M, n = 7) or Cavα1.2 Q428E (Q428E, n = 6) at baseline and 24 h after treatment with 1.3 μM R7W-MP (T320M + R7W-MP, n = 12; Q428E + R7W-MP, n = 8). (B) Single dots and histograms (average) are showing ICa measured at a test pulse of +10 mV for all the above-mentioned experimental conditions. *p < 0.05, **p < 0.01 (Kruskal-Wallis test). (C) Graphical representation of ICa activation-curves (g/gmax) for WT (V1/2 = −10.34 ± 1.28, slope = 7.08 ± 0.57), T320M (V1/2 = −7.08 ± 1.77, slope = 7.29 ± 0.46), and Q428E (V1/2 = −7.10 ± 1.64, slope = 6.59 ± 0.25). Kinetic curves did not change after R7W-MP treatment (V1/2 = −8.28 ± 1.32 and slope = 6.78 ± 0.28; V1/2 = −6.91 ± 0.91 and slope = 6.66 ± 0.37 for T320M + R7W-MP and Q428E + R7W-MP, respectively). [file Image_1.jpeg]

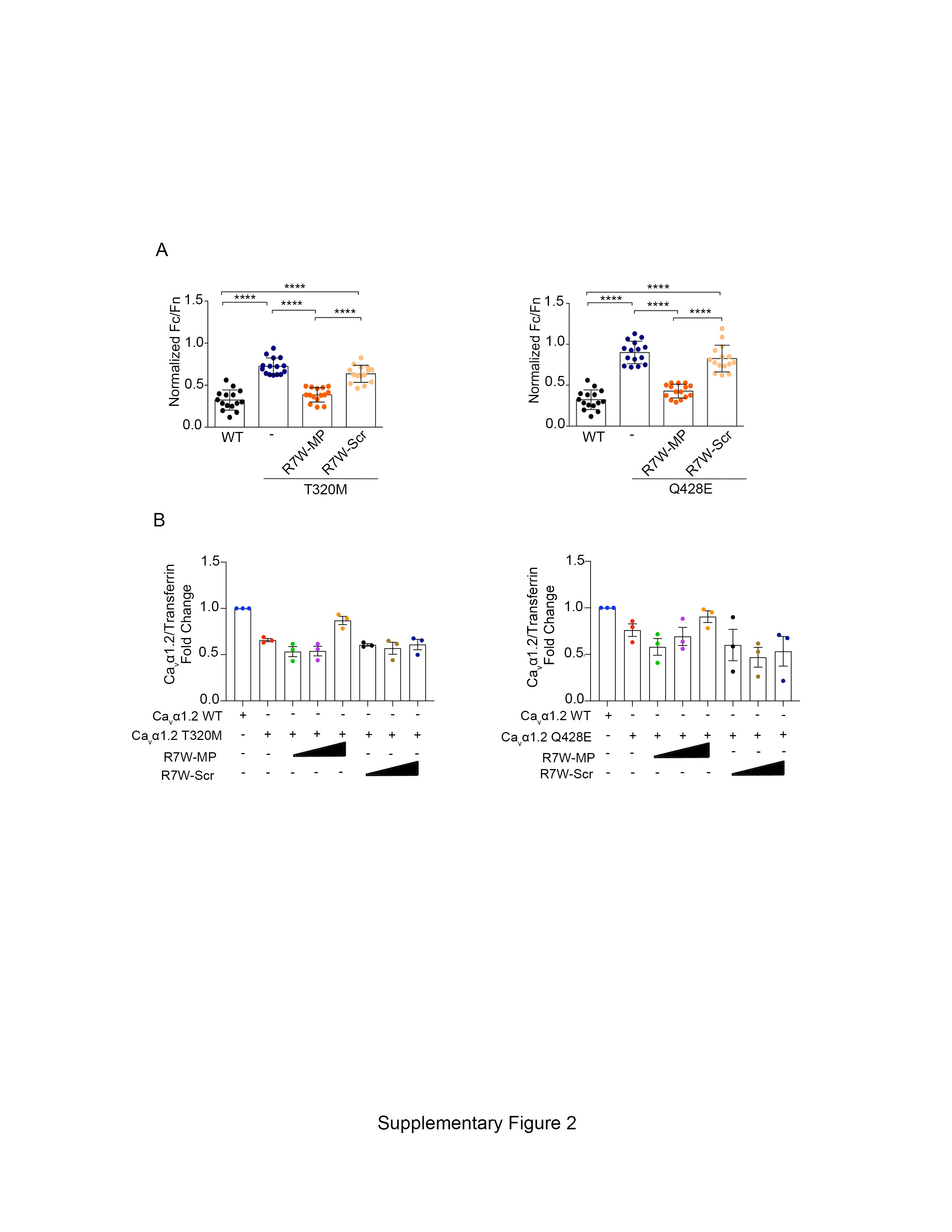

Supplement: SUPPLEMENTARY FIGURE 2 — R7W-MP corrects LTCC density at the plasma membrane in Cavα1.2 mutant-transfected HEK293 cells. (A) Quantification of the fluorescence signal in the cytoplasm and the membrane of the cells (n = 15) in Figure 4A. Fc, cytoplasmic fluorescence; Fm, membrane fluorescence. *** p < 0.001 (one-way ANOVA). (B) Graphical representation of densitometry of Western blot for Cavα1.2 in the cell surface biotinylation assays in Figure 4B. (Kruskal-Wallis test). [file Image_2.jpeg]
